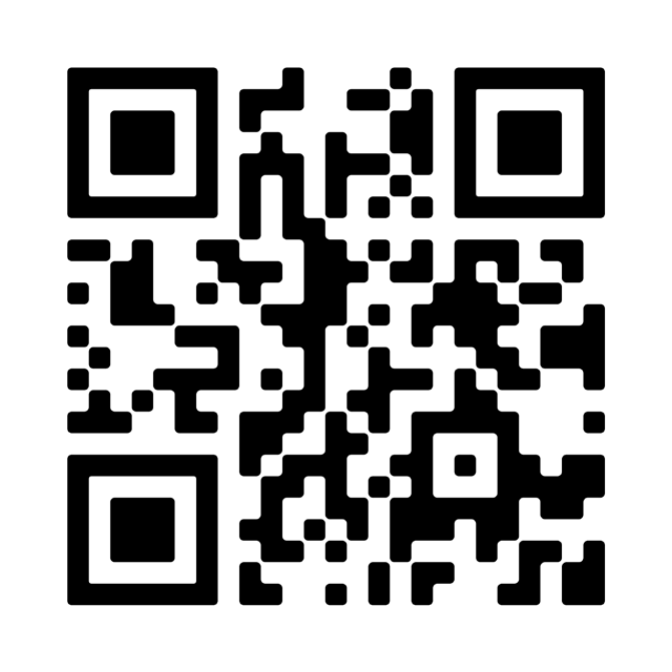# A new surgical approach for fertility-sparing management of diffuse endometrial G2 endometrioid adenocarcinoma: a step-by-step technique

**DOI:** 10.52054/FVVO.15.1.058

**Published:** 2023-03-31

**Authors:** U Catena, M Mirandola, F.M. Capomacchia, F Fanfani, G Scambia

**Affiliations:** Dipartimento di Scienze della Salute della Donna, del Bambino e di Sanità Pubblica, Fondazione Policlinico Universitario A. Gemelli IRCCS, U.O.C. di Ginecologia Oncologica, Rome, Italy; Dipartimento Scienze della Vita e Sanità Pubblica, Università Cattolica del Sacro Cuore, Rome, Italy

**Keywords:** Endometrial cancer, fertility-sparing, moderately differentiated (G2) endometrial cancer, hysteroscopy

## Abstract

**Background:**

4% of endometrial cancers are diagnosed in young women and 70% are nulliparous. Preserve fertility in these patients is of major interest. It is demonstrated that hysteroscopic resection of focal well-differentiated endometrioid adenocarcinoma, followed by progestins achieve a complete response rate of 95.3%. Recently, fertility-sparing treatment was proposed also in case of moderately differentiated endometrioid tumors, with a relatively high remission rate.

**Objectives:**

To show a new hysteroscopic approach, in case of fertility-sparing treatment of diffuse endometrial G2 endometrioid adenocarcinoma.

**Materials and Methods:**

Stepwise demonstration of the technique with narrated video footage for the fertility- sparing management of diffuse endometrial G2 endometrioid adenocarcinoma, combining the use of a 15 Fr bipolar miniresectoscope and ‘three-steps’ resection technique (Karl Storz, Tuttlingen, Germany) with a Tissue Removal Device (TRD) (Truclear Elite Mini, Medtronic).

**Main Outcome Measures:**

negative hysteroscopic assessment and endometrial biopsies at three and six months.

**Results:**

Normal endometrial cavity and negative biopsies

**Conclusion:**

Combined hysteroscopic technique, in case of diffuse endometrial G2 endometrioid adenocarcinoma, followed by double progestin therapy (Levonorgestrel releasing Intrauterine Device + Megestrole Acetate 160 mg/ daily), may be associated with higher complete response rate; the use of TRD to complete the resection near tubal ostia may reduce risk of post-operative intrauterine adhesions and improve reproductive outcomes.

**What is new?:**

A novel, fertility-sparing surgical approach for diffuse endometrial G2 endometroid adenocarcinoma.

## Learning Objective

To show a new hysteroscopic approach, combining the use of a 15 Fr bipolar miniresectoscope (Karl Storz, Tuttlingen, Germany) with a Tissue Removal Device (TRD) (Truclear Elite Mini, Medtronic) followed by double progestin therapy (Levonorgestrel releasing Intrauterine Device + Megestrole Acetate 160 mg/daily), for fertility-sparing treatment of diffuse endometrial G2 endometrioid adenocarcinoma, stage I at preoperative imaging assessment.

## Introduction

4% of women diagnosed with EC are younger than 40 years old, and 70% of these women are nulliparous (Siegel et al., 2006; Kataoka et al., 2014). Preservation of fertility is of great significance to many of these patients. It has been demonstrated that hysteroscopic resection of focal well-differentiated (Grade 1) endometrioid adenocarcinoma, followed by progestins achieve a complete response rate of 95.3% (Falcone et al., 2017; Falcone et al., 2020; Mazzon et al., 2010).

The hysteroscopic resection technique, the so- called “three steps” technique, was firstly described by Mazzon et al. (2005) to treat focal Grade 1 (G1) EC without myometrial invasion. Some years later, Giampaolino et al. (2019) described the hysteroscopic technique to treat diffuse atypical endometrial hyperplasia (AEH), consisting in superficial endometrial resection, preserving the basal layer of the endometrium. Recently, fertility- sparing treatment was proposed also in case of moderately-differentiated endometrioid (grading G2) tumours, with a relatively high remission rate (Giampaolino et al., 2022; Koskas et al., 2011; Park et al., 2013; Vitale et al., 2017).

Complete surgical resection of endometrial cancer prior to starting progestin therapy seems to improve the complete response rate. Although results are encouraging, patients who are candidates for fertility-sparing treatment must be referred to specialised centres, using well-defined protocols and close follow-up (Concin et al., 2021; Gallo et al., 2021).

## Patients and methods

A 38-year-old patient was referred to our oncological centre with a histological diagnosis of moderately differentiated (G2) endometrial endometrioid adenocarcinoma. She strongly wanted to preserve her fertility. Preoperative imaging (ultrasound, magnetic resonance, and CT- scan) showed a disease limited to the endometrium, with diffuse pathological endometrial tissue limited to the endometrial basal layer. There was no suspicious of ovarian pathology and no evidence of cervical extension and/or pelvic or para-aortic lymphadenopathies leading to a diagnosis of radiological stage I endometrial cancer. After adequate counselling, a fertility-sparing approach was agreed.

This is a stepwise demonstration of a step-by- step hysteroscopic procedure for the fertility- sparing management of diffuse endometrial G2 endometrioid adenocarcinoma with narrated video footage.

The patient underwent the hysteroscopic procedure in an outpatient setting under conscious sedation (Level 3(b) pain management, as achieved with i.v. midazolam 10 milligrams and fentanyl 100 micrograms) (Carugno et al., 2021; Carugno et al., 2022). Hysteroscopy was performed using a vaginoscopic approach and saline solution was used as distension medium.

A 15 Fr Miniresectoscope was used to remove the main lesions according to the so called “three steps” technique, described by Mazzon et al., 2010: 1) resection of the lesion; 2) resection of the endometrium adjacent to the lesion (4–5 mm outside); 3) resection of the myometrium underlying the lesion (3–4 mm). The Tissue Removal Device (TRD) was then used to complete the resection of the pathological endometrium in difficult areas such as the tubal ostia.

At the end of the procedure, all the diffuse pathological tissue was removed. No complications were documented, and patient was discharged 3 hours after the procedure. The estimated blood loss was 20mL.

Double progestin therapy was then administered, through a levonorgestrel-releasing intrauterine device and oral megestrol acetate 160 mg/daily.

A three and six months hysteroscopic control showed a normal uterine cavity with negative endometrial biopsies.

## Discussion

Combined hysteroscopic resection with 15Fr bipolar miniresectoscope and TRD allowed a complete resection of the pathological tissue in case of diffuse endometrial G2 endometrioid adenocarcinoma.

The demonstrated hysteroscopic technique requires advanced surgical skills and it ought to be performed in a specialised oncological referral centre. Conservative management of moderate- high grade disease should be considered with caution, given that there are few studies and limited numbers of cases of fertility-sparing treatment in these patients (Giampaolino et al., 2022).

According to the more recent guidelines, fertility-sparing treatment should be considered only in case of atypical endometrial hyperplasia or grade 1 endometrioid carcinoma without myometrial invasion (Concin et al., 2021). In these patients, hysteroscopic resection of the tumour prior to starting progestin therapy has been shown better results in terms of complete response rate and recurrence rate compared to the progestin therapy alone (Gallo et al., 2021).

The hysteroscopic surgical techniques described to treat these women vary according to the diffusion of the lesions inside the uterine cavity. Mazzon et al. (2005) firstly described the so called “three steps” technique to treat focal lesions. Giampaolino et al. (2019), described a novel hysteroscopic technique to remove diffuse atypical endometrial hyperplasia. This technique consisted in the use of a 26 Fr resectoscope to remove the pathological endometrium while preserving the basal layer of the endometrial glands. The use of tissue removal devices (TRD) has been widely described in case of benign intracavitary pathologies and associated with lower risk of intrauterine adhesions (IUAs) formation (Hamerlynck et al., 2016)

In light of these studies, we proposed a new hysteroscopic approach to treat diffuse endometrial G2 endometrioid adenocarcinoma. A 15 Fr bipolar miniresectoscope was used to remove the main lesions according to the Mazzon “three steps” technique (Mazzon et al., 2010) and a TRD was used to complete the resection of the pathological tissue in difficult areas, such as the tubal ostia. The demonstrated surgical technique, followed by the administration of double progestin therapy (Levonorgestrel releasing Intrauterine Device and oral megestrol acetate), may be associated with higher complete response rate. The use of TRD to complete the resection may reduce the risk of electrosurgery related complications such as post- operative IUAs formation. Fertility post-operative outcomes may therefore benefit from the reduction of IUAs.

Although this result is encouraging, patients who are candidates for this approach ought to be referred to specialised centres which use well-defined protocols, offer detailed counselling regarding the possible risks and ensure close follow-up (Concin et al., 2021; Gallo et al., 2021).

## Conclusions

Combined hysteroscopic resection with 15 Fr bipolar miniresectoscope and TRD, in the case of diffuse endometrial G2 endometrioid adenocarcinoma, followed by the administration of double progestin therapy (Levonorgestrel releasing Intrauterine Device and oral megestrol acetate), may be associated with a higher complete response rate. Complete resection of the tumour, even in difficult areas, is made possible using a TRD, which reduces the use of electrosurgery and the subsequent risk of post-operative intrauterine adhesions. This could lead to better reproductive outcomes in these patients. Further studies are needed to refine the indications and the surgical technique.

## Video scan (read QR)

https://vimeo.com/762169020/130a2b6bca

**Figure:**